# Bis(trimethyl­phenyl­ammonium) hexa­[bromido/chlorido(0.792/0.208)]stannate(IV)

**DOI:** 10.1107/S160053681000680X

**Published:** 2010-03-03

**Authors:** Kong Mun Lo, Seik Weng Ng

**Affiliations:** a Department of Chemistry, University of Malaya, 50603 Kuala Lumpur, Malaysia

## Abstract

In the title mol­ecular salt, [C_6_H_5_(CH_3_)_3_N]_2_[SnBr_4.75_Cl_1.25_], the Sn^IV^ atom (site symmetry 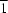) adopts an octa­hedral coordination geometry. The Br and Cl atoms are disordered over three sites in 0.7415 (13):0.2585 (14), 0.8514 (14):0.1486 (14) and 0.7821 (14):0.2179 (14) ratios.

## Related literature

For the crystal structures of other ammonium hexa­bromidostannates(IV): see: Al-Far & Ali (2007 ▶); Al-Far *et al.* (2009 ▶); Ali *et al.* (2007 ▶); Howie *et al.* (2009 ▶).
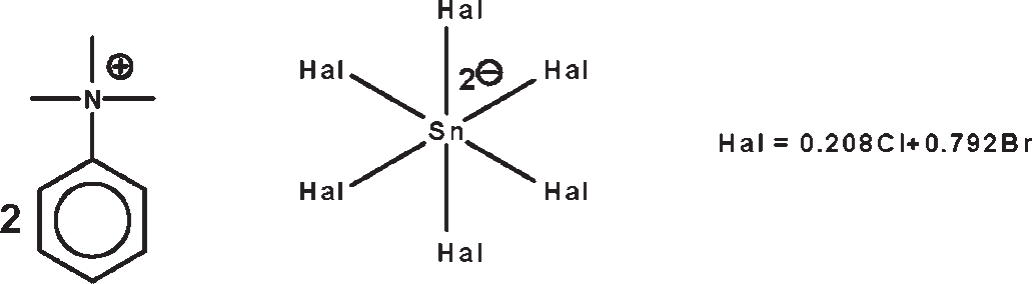


## Experimental

### 

#### Crystal data

(C_9_H_14_N)_2_[SnBr_4.75_Cl_1.25_]
*M* *_r_* = 815.00
Monoclinic, 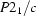

*a* = 8.8003 (1) Å
*b* = 10.6362 (2) Å
*c* = 14.2869 (2) Å
β = 104.433 (1)°
*V* = 1295.07 (3) Å^3^
*Z* = 2
Mo *K*α radiation
μ = 8.45 mm^−1^
*T* = 293 K
0.30 × 0.30 × 0.20 mm

#### Data collection

Bruker SMART APEX diffractometer
Absorption correction: multi-scan (*SADABS*; Sheldrick, 1996 ▶) *T*_min_ = 0.186, *T*_max_ = 0.283
12094 measured reflections
2974 independent reflections
2507 reflections with *I* > 2σ(*I*)
*R*_int_ = 0.023

#### Refinement

*R*[*F* ^2^ > 2σ(*F* ^2^)] = 0.021
*wR*(*F* ^2^) = 0.052
*S* = 1.01
2974 reflections
133 parameters
5 restraints
H-atom parameters constrained
Δρ_max_ = 0.38 e Å^−3^
Δρ_min_ = −0.58 e Å^−3^

### 

Data collection: *APEX2* (Bruker, 2009 ▶); cell refinement: *SAINT* (Bruker, 2009 ▶); data reduction: *SAINT*; program(s) used to solve structure: *SHELXS97* (Sheldrick, 2008 ▶); program(s) used to refine structure: *SHELXL97* (Sheldrick, 2008 ▶); molecular graphics: *X-SEED* (Barbour, 2001 ▶); software used to prepare material for publication: *publCIF* (Westrip, 2010 ▶).

## Supplementary Material

Crystal structure: contains datablocks global, I. DOI: 10.1107/S160053681000680X/hb5336sup1.cif

Structure factors: contains datablocks I. DOI: 10.1107/S160053681000680X/hb5336Isup2.hkl

Additional supplementary materials:  crystallographic information; 3D view; checkCIF report

## Figures and Tables

**Table 1:** Selected bond lengths (Å)

Sn1—Br1	2.5630 (3)
Sn1—Br2	2.5886 (3)
Sn1—Br3	2.5874 (3)

**Fig. 1.**
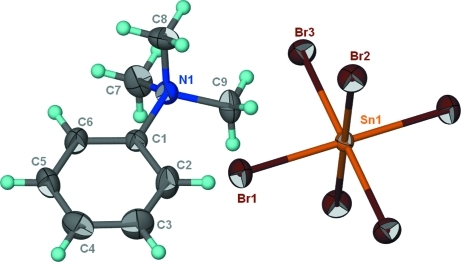
The molecular structure of (I) at the 50% probability level. Hydrogen atoms are drawn as spheres of arbitrary radius. The bromine atoms are disordered with respect to the chlorine atoms.

## supplementary crystallographic information

### Experimental

Tribenzyltin chloride (0.34 g, 1 mmol) and trimethylphenylammonium tribromide (0.38 g, 1 mmol) were heated in ethanol (50 ml) for 1 hour. After filtering of the reaction mixture, yellow blocks of (I) were obtained upon slow evaporation of the filtrate.  The crystal structure indicated that all the organic groups bonded to tin in the reactant were cleaved by the tribromide anion.

### Refinement

Hydrogen atoms were placed at calculated positions (C–H 0.93–0.96 Å) and were treated as riding on their parent atoms, with *U*(H) set to 1.2–1.5 times *U*_eq_(C). The initial refinement that assumed the halogens were only bromine atoms led to a difference Fourier with a large peak near Sn1 and a deep hole near Br1. The *R*-index was 0.0367.

The three halogen atoms were then refined as a mixture of chlorine and bromine. For each site, the displacement factor of the bromine and chlorine occupants were restrained to be identical. The refinement gave nearly 2.375 bromine and 0.625 chlorine atoms, and the difference Fourier was diffuse.

### Crystal data

**Table:**

(C_9_H_14_N)_2_[SnBr_4.75_Cl_1.25_]	*F*(000) = 775
*M**_r_* = 815.00	*D*_x_ = 2.090 Mg m^−^^3^
Monoclinic, *P*2_1_/*c*	Mo *K*α radiation, λ = 0.71073 Å
Hall symbol: -P 2ybc	Cell parameters from 4823 reflections
*a* = 8.8003 (1) Å	θ = 2.4–28.2°
*b* = 10.6362 (2) Å	µ = 8.45 mm^−^^1^
*c* = 14.2869 (2) Å	*T* = 293 K
β = 104.433 (1)°	Block, yellow
*V* = 1295.07 (3) Å^3^	0.30 × 0.30 × 0.20 mm
*Z* = 2	

### Data collection

**Table:**

Bruker SMART APEX diffractometer	2974 independent reflections
Radiation source: fine-focus sealed tube	2507 reflections with *I* > 2σ(*I*)
graphite	*R*_int_ = 0.023
ω scans	θ_max_ = 27.5°, θ_min_ = 2.4°
Absorption correction: multi-scan (*SADABS*; Sheldrick, 1996)	*h* = −11→11
*T*_min_ = 0.186, *T*_max_ = 0.283	*k* = −13→13
12094 measured reflections	*l* = −18→18

### Refinement

**Table:**

Refinement on *F*^2^	Primary atom site location: structure-invariant direct methods
Least-squares matrix: full	Secondary atom site location: difference Fourier map
*R*[*F*^2^ > 2σ(*F*^2^)] = 0.021	Hydrogen site location: inferred from neighbouring sites
*wR*(*F*^2^) = 0.052	H-atom parameters constrained
*S* = 1.01	*w* = 1/[σ^2^(*F*_o_^2^) + (0.0273*P*)^2^ + 0.3311*P*] where *P* = (*F*_o_^2^ + 2*F*_c_^2^)/3
2974 reflections	(Δ/σ)_max_ = 0.001
133 parameters	Δρ_max_ = 0.38 e Å^−^^3^
5 restraints	Δρ_min_ = −0.58 e Å^−^^3^

### Fractional atomic coordinates and isotropic or equivalent isotropic displacement parameters (Å^2^)

**Table:**

	*x*	*y*	*z*	*U*_iso_*/*U*_eq_	Occ. (<1)
Sn1	0.5000	0.5000	0.5000	0.02940 (7)	
Br1	0.79909 (3)	0.50477 (3)	0.52754 (2)	0.04523 (11)	0.7415 (13)
Br2	0.50568 (4)	0.71415 (3)	0.58689 (2)	0.04665 (11)	0.8514 (14)
Br3	0.53087 (4)	0.38474 (3)	0.66315 (2)	0.04326 (11)	0.7821 (14)
Cl1	0.79909 (3)	0.50477 (3)	0.52754 (2)	0.04523 (11)	0.2585 (14)
Cl2	0.50568 (4)	0.71415 (3)	0.58689 (2)	0.04665 (11)	0.1486 (14)
Cl3	0.53087 (4)	0.38474 (3)	0.66315 (2)	0.04326 (11)	0.2179 (14)
N1	0.8193 (2)	0.0359 (2)	0.70182 (15)	0.0404 (5)	
C1	0.9413 (3)	0.0799 (2)	0.65257 (17)	0.0354 (5)	
C2	0.8999 (3)	0.1456 (3)	0.5678 (2)	0.0586 (8)	
H2	0.7949	0.1610	0.5382	0.070*	
C3	1.0162 (4)	0.1887 (3)	0.5271 (3)	0.0689 (10)	
H3	0.9879	0.2330	0.4692	0.083*	
C4	1.1700 (4)	0.1687 (3)	0.5682 (2)	0.0564 (8)	
H4	1.2466	0.1978	0.5390	0.068*	
C5	1.2101 (4)	0.1053 (3)	0.6533 (2)	0.0636 (9)	
H5	1.3155	0.0919	0.6831	0.076*	
C6	1.0965 (3)	0.0603 (3)	0.6963 (2)	0.0566 (8)	
H6	1.1253	0.0170	0.7546	0.068*	
C7	0.8529 (4)	−0.0962 (3)	0.7393 (3)	0.0619 (9)	
H7A	0.8527	−0.1518	0.6864	0.093*	
H7B	0.7735	−0.1221	0.7708	0.093*	
H7C	0.9537	−0.0988	0.7847	0.093*	
C8	0.8202 (4)	0.1222 (3)	0.7851 (2)	0.0636 (9)	
H8A	0.9203	0.1176	0.8310	0.095*	
H8B	0.7393	0.0973	0.8156	0.095*	
H8C	0.8015	0.2069	0.7618	0.095*	
C9	0.6576 (3)	0.0366 (3)	0.6359 (2)	0.0567 (8)	
H9A	0.6272	0.1215	0.6175	0.085*	
H9B	0.5852	0.0008	0.6689	0.085*	
H9C	0.6569	−0.0122	0.5793	0.085*	

### Atomic displacement parameters (Å^2^)

**Table:**

	*U*^11^	*U*^22^	*U*^33^	*U*^12^	*U*^13^	*U*^23^
Sn1	0.02718 (11)	0.02958 (12)	0.03066 (12)	−0.00216 (9)	0.00577 (8)	−0.00056 (9)
Br1	0.02788 (16)	0.0596 (2)	0.04703 (19)	−0.00311 (13)	0.00721 (13)	0.00024 (14)
Br2	0.05446 (19)	0.03536 (16)	0.04899 (19)	−0.00321 (13)	0.01072 (14)	−0.00974 (12)
Br3	0.04800 (18)	0.04521 (18)	0.03699 (17)	0.00217 (13)	0.01141 (13)	0.00745 (13)
Cl1	0.02788 (16)	0.0596 (2)	0.04703 (19)	−0.00311 (13)	0.00721 (13)	0.00024 (14)
Cl2	0.05446 (19)	0.03536 (16)	0.04899 (19)	−0.00321 (13)	0.01072 (14)	−0.00974 (12)
Cl3	0.04800 (18)	0.04521 (18)	0.03699 (17)	0.00217 (13)	0.01141 (13)	0.00745 (13)
N1	0.0381 (11)	0.0413 (12)	0.0425 (12)	0.0010 (9)	0.0116 (9)	0.0063 (10)
C1	0.0365 (13)	0.0337 (12)	0.0367 (13)	−0.0019 (10)	0.0107 (10)	−0.0008 (10)
C2	0.0461 (16)	0.064 (2)	0.0621 (19)	0.0077 (15)	0.0071 (14)	0.0280 (16)
C3	0.075 (2)	0.071 (2)	0.064 (2)	−0.0009 (18)	0.0238 (18)	0.0325 (18)
C4	0.0603 (19)	0.0526 (18)	0.064 (2)	−0.0082 (15)	0.0306 (16)	0.0018 (15)
C5	0.0394 (15)	0.086 (2)	0.068 (2)	0.0031 (16)	0.0184 (15)	0.0090 (18)
C6	0.0422 (15)	0.081 (2)	0.0456 (16)	0.0065 (15)	0.0101 (13)	0.0173 (16)
C7	0.0571 (18)	0.0496 (18)	0.082 (2)	0.0043 (14)	0.0235 (17)	0.0281 (16)
C8	0.069 (2)	0.077 (2)	0.0519 (18)	0.0022 (17)	0.0294 (16)	−0.0079 (16)
C9	0.0356 (14)	0.0623 (19)	0.068 (2)	−0.0023 (13)	0.0049 (14)	0.0087 (16)

### Geometric parameters (Å, °)

**Table:**

Sn1—Cl1^i^	2.5630 (3)	C3—C4	1.352 (4)
Sn1—Br1^i^	2.5630 (3)	C3—H3	0.9300
Sn1—Br1	2.5630 (3)	C4—C5	1.357 (5)
Sn1—Cl3^i^	2.5874 (3)	C4—H4	0.9300
Sn1—Br3^i^	2.5874 (3)	C5—C6	1.383 (4)
Sn1—Br2	2.5886 (3)	C5—H5	0.9300
Sn1—Br3	2.5874 (3)	C6—H6	0.9300
Sn1—Br2^i^	2.5886 (3)	C7—H7A	0.9600
Sn1—Cl2^i^	2.5886 (3)	C7—H7B	0.9600
N1—C8	1.501 (4)	C7—H7C	0.9600
N1—C1	1.498 (3)	C8—H8A	0.9600
N1—C9	1.498 (3)	C8—H8B	0.9600
N1—C7	1.506 (4)	C8—H8C	0.9600
C1—C6	1.368 (4)	C9—H9A	0.9600
C1—C2	1.367 (4)	C9—H9B	0.9600
C2—C3	1.375 (4)	C9—H9C	0.9600
C2—H2	0.9300		
			
Cl1^i^—Sn1—Br1^i^	0.00 (2)	C8—N1—C7	109.1 (2)
Cl1^i^—Sn1—Br1	180.000 (15)	C1—N1—C7	111.0 (2)
Br1^i^—Sn1—Br1	180.000 (15)	C9—N1—C7	107.4 (2)
Cl1^i^—Sn1—Cl3^i^	89.879 (10)	C6—C1—C2	119.8 (2)
Br1^i^—Sn1—Cl3^i^	89.879 (10)	C6—C1—N1	119.2 (2)
Br1—Sn1—Cl3^i^	90.121 (10)	C2—C1—N1	120.8 (2)
Cl1^i^—Sn1—Br3^i^	89.879 (10)	C1—C2—C3	118.9 (3)
Br1^i^—Sn1—Br3^i^	89.879 (10)	C1—C2—H2	120.6
Br1—Sn1—Br3^i^	90.121 (10)	C3—C2—H2	120.6
Cl3^i^—Sn1—Br3^i^	0.000 (6)	C4—C3—C2	122.3 (3)
Cl1^i^—Sn1—Br3	90.121 (10)	C4—C3—H3	118.9
Br1^i^—Sn1—Br3	90.121 (10)	C2—C3—H3	118.9
Br1—Sn1—Br3	89.879 (10)	C3—C4—C5	118.4 (3)
Cl3^i^—Sn1—Br3	180.0	C3—C4—H4	120.8
Br3^i^—Sn1—Br3	180.0	C5—C4—H4	120.8
Cl1^i^—Sn1—Br2	89.277 (10)	C4—C5—C6	121.0 (3)
Br1^i^—Sn1—Br2	89.277 (10)	C4—C5—H5	119.5
Br1—Sn1—Br2	90.723 (10)	C6—C5—H5	119.5
Cl3^i^—Sn1—Br2	90.014 (10)	C1—C6—C5	119.6 (3)
Br3^i^—Sn1—Br2	90.014 (10)	C1—C6—H6	120.2
Br3—Sn1—Br2	89.986 (10)	C5—C6—H6	120.2
Cl1^i^—Sn1—Br2^i^	90.723 (10)	N1—C7—H7A	109.5
Br1^i^—Sn1—Br2^i^	90.723 (10)	N1—C7—H7B	109.5
Br1—Sn1—Br2^i^	89.277 (10)	H7A—C7—H7B	109.5
Cl3^i^—Sn1—Br2^i^	89.986 (10)	N1—C7—H7C	109.5
Br3^i^—Sn1—Br2^i^	89.986 (10)	H7A—C7—H7C	109.5
Br3—Sn1—Br2^i^	90.014 (10)	H7B—C7—H7C	109.5
Br2—Sn1—Br2^i^	180.0	N1—C8—H8A	109.5
Cl1^i^—Sn1—Cl2^i^	90.723 (10)	N1—C8—H8B	109.5
Br1^i^—Sn1—Cl2^i^	90.723 (10)	H8A—C8—H8B	109.5
Br1—Sn1—Cl2^i^	89.277 (10)	N1—C8—H8C	109.5
Cl3^i^—Sn1—Cl2^i^	89.986 (10)	H8A—C8—H8C	109.5
Br3^i^—Sn1—Cl2^i^	89.986 (10)	H8B—C8—H8C	109.5
Br3—Sn1—Cl2^i^	90.014 (10)	N1—C9—H9A	109.5
Br2—Sn1—Cl2^i^	180.0	N1—C9—H9B	109.5
Br2^i^—Sn1—Cl2^i^	0.000 (6)	H9A—C9—H9B	109.5
C8—N1—C1	108.6 (2)	N1—C9—H9C	109.5
C8—N1—C9	108.1 (2)	H9A—C9—H9C	109.5
C1—N1—C9	112.5 (2)	H9B—C9—H9C	109.5
			
C8—N1—C1—C6	73.7 (3)	N1—C1—C2—C3	177.3 (3)
C9—N1—C1—C6	−166.6 (3)	C1—C2—C3—C4	−0.3 (6)
C7—N1—C1—C6	−46.2 (4)	C2—C3—C4—C5	−0.8 (6)
C8—N1—C1—C2	−102.3 (3)	C3—C4—C5—C6	1.0 (5)
C9—N1—C1—C2	17.4 (4)	C2—C1—C6—C5	−1.2 (5)
C7—N1—C1—C2	137.8 (3)	N1—C1—C6—C5	−177.3 (3)
C6—C1—C2—C3	1.4 (5)	C4—C5—C6—C1	0.0 (5)

Symmetry codes: (i) −*x*+1, −*y*+1, −*z*+1.
